# Williams-Beuren Syndrome: A Clinical Study of 55 Brazilian Patients and the Diagnostic Use of MLPA

**DOI:** 10.1155/2015/903175

**Published:** 2015-05-18

**Authors:** Rachel Sayuri Honjo, Roberta Lelis Dutra, Erika Arai Furusawa, Evelin Aline Zanardo, Larissa Sampaio de Athayde Costa, Leslie Domenici Kulikowski, Debora Romeo Bertola, Chong Ae Kim

**Affiliations:** ^1^Clinical Genetics Unit, Instituto da Criança, Hospital das Clínicas, Faculdade de Medicina, Universidade de São Paulo, 05403-000 São Paulo, SP, Brazil; ^2^Department of Pathology, Laboratório de Citogenômica, LIM 03, Hospital das Clínicas, Faculdade de Medicina, Universidade de São Paulo, 05403-000 São Paulo, SP, Brazil; ^3^Department of Nephrology, Instituto da Criança, Hospital das Clínicas, Faculdade de Medicina, Universidade de São Paulo, 05403-000 São Paulo, SP, Brazil

## Abstract

Williams-Beuren syndrome (WBS) is a genetic disease caused by a microdeletion in the 7q11.23 region. It is characterized by congenital heart disease, mainly supravalvular aortic stenosis, mental retardation, mild short stature, facial dysmorphisms, and variable abnormalities in different systems. *Objectives*. To report the clinical findings of 55 Brazilian patients confirmed by multiplex ligation-dependent probe amplification (MLPA). *Methods*. Patients were followed up for 4 years at the Genetics Unit of the Instituto da Criança of the Hospital das Clínicas, FMUSP, Brazil. A kit specific for WBS was used to detect the 7q11.23 microdeletion. *Results*. Two patients with negative FISH results had positive MLPA results for WBS. The characteristics of the patients with the deletion were as follows: typical WBS facies (98.2%), neuropsychomotor delay (98.2%), hypersocial behavior (94.5%), hyperacusis (94.5%), and congenital heart disease (81.8%). *Conclusions*. MLPA was effective in detecting the microdeletion in the 7q11.23 region to confirm the diagnosis of WBS. MLPA was also able to confirm the diagnosis of WBS in two patients with typical clinical characteristics but negative FISH results. Thus, MLPA is a promising method in the diagnostic investigation of WBS. WBS is a multisystemic disorder and therefore requires multidisciplinary care and specific follow-up to prevent complications.

## 1. Introduction

Williams-Beuren syndrome (WBS) is a genetic multisystemic disease characterized by congenital heart disease, mainly supravalvular aortic stenosis (SVAS), mental retardation, mild short stature, facial dysmorphisms, and variable abnormalities in the genitourinary, endocrinological, ophthalmological, and skeletal systems [1, 2]. The incidence is estimated to be 1 in 20,000 live births [3], but some authors report a prevalence of approximately 1 in 7,500 [4].

The typical facial dysmorphisms found in WBS are as follows: high forehead, medial broadening of the eyebrows, periorbital fullness, depressed nasal bridge, malar hypoplasia, thick lips, and long nasolabial philtrum [1, 2, 5]. Short stature is common [6] but not severe.

Several studies report that patients with WBS have unique cognitive and behavioral profiles, with characteristic dissociations among different domains, such as better skills in language and deficits in motor and visuospatial activities [7–9]. Patients also have characteristic hypersocial behavior, even with strangers [10, 11].

WBS is caused by a 1-2 Mb microdeletion in 7q11.23, a region that contains 28 genes [12]. Approximately 90% of WBS patients have a 1.55 Mb microdeletion and 8% have a 1.84 Mb microdeletion. These are considered “typical” WBS microdeletions. Microdeletions larger than 1.84 Mb or smaller than 1.55 Mb are termed “atypical,” are often associated with atypical clinical manifestations, and occur in only 2% of cases [13]. The recognition and description of these cases have been very helpful for genotype-phenotype correlation studies. Whether the parental origin of the microdeletion has any impact on the phenotype of the patient remains under debate [14–16].

WBS is generally sporadic [16–18], is caused by de novo deletions, and has a recurrence risk lower than 5% [19–21]. A few cases of vertical transmission have been reported [12, 22–24]. People with microinversions of 1.5–1.9 Mb in the WBS critical region are predisposed to having children with WBS [25–27].

The chromosomal region 7q11.23 comprises a region of approximately 1.2 Mb of single copy genes and three blocks of low copy repeat sequences. Due to the high similarity of those blocks, nonallelic homologous recombination is possible and can result in microdeletion or microduplication within the region [12, 16, 28].

Although individuals with WBS present with a highly characteristic phenotypic profile, the diagnosis of WBS is often confirmed by molecular testing. Currently, the microdeletion in the 7q11.23 region can be detected by several methods, including fluorescence in situ hybridization (FISH), polymorphic microsatellite markers, chromosomal microarray analysis (CMA), and* multiplex ligation-dependent probe amplification* (MLPA).

Some studies have shown that MLPA is an alternative to FISH, which is the current gold-standard method for diagnosing WBS. Cho et al. [29] found concordant results in four patients using both techniques.

Here, we report the clinical findings of 55 Brazilian patients with WBS confirmed by MLPA.

## 2. Methods

The patients were evaluated at the Genetics Unit of the Instituto da Criança of the Hospital das Clínicas da Faculdade de Medicina da Universidade de São Paulo (ICr, HCFMUSP), São Paulo, Brazil. The study was approved by the local Ethics Board, and informed consent form was obtained from all families. All patients were evaluated and followed up by a single examiner over a period of 4 years (2008–2011). Clinical and laboratory data were collected following a protocol that included the following: anamnesis, physical examination, cardiovascular assessment (arterial pressure and echocardiogram), urinary tract evaluation (renal ultrasonogram, BUN, and creatinine), plasmatic and urinary calcium, thyroid function tests, and referral to specialists for baseline and/or follow-up evaluations (ophthalmology, cardiology, nephrology, psychiatry, and endocrinology, among others).

DNA was extracted from peripheral blood by the salting-out method [30]. MLPA analyses were performed using kit P029 from MRC Holland (Amsterdam, Netherlands), following the manufacturer's instructions. This kit contained probes of genes mapped to the WBS critical region (*ELN*,* CLIP2*,* LIMK1*,* TBL2*,* STX1A*,* RFC2*,* FZD9*, and* FKBP6*) and controls. Data were analyzed using the GeneMarker software.

## 3. Results

**Figure 1:**
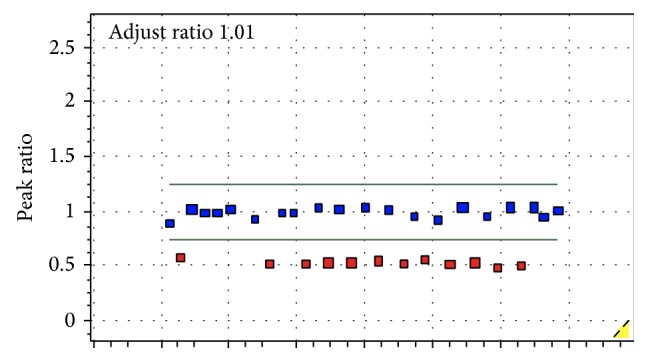
MLPA showing deletion of the probes in the 7q11.23 region (red squares).

Fifty-five patients (34 males and 21 females) with clinical diagnoses of WBS were evaluated. The age at diagnosis ranged from 2 to 30 years old (median = 14 years). MLPA confirmed the microdeletion in 7q11.23 (Figure 1).

**Figure 2:**
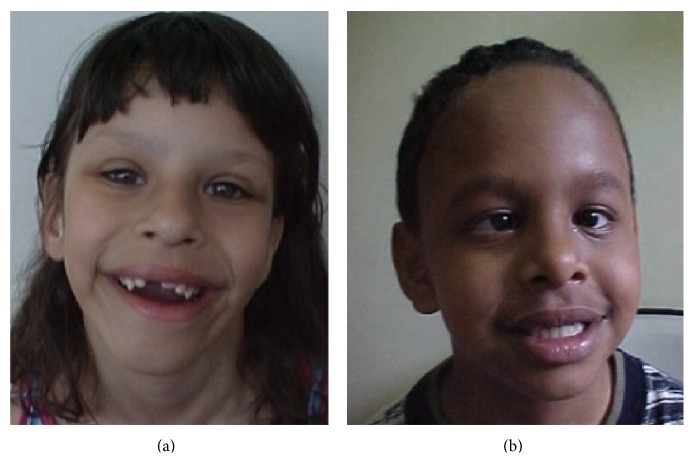
Patients with positive MLPA but negative FISH results for WBS.

FISH results were available for 18 of the 55 patients; the results were positive for all but two. MLPA confirmed the diagnosis in these two patients. The first patient was a girl (Figure 2(a)), born at term, after an uneventful pregnancy except for the use of penicillin by the mother in the 2nd month. The mother had had one previous spontaneous abortion and had another child with autism. The patient was born by cesarean section due to fetal bradycardia, with 2620 g and 47 cm. She had meningitis within 21 days and presented with neuromotor development delay (sat after 8 months, walked at 2 years of age, and spoke first words at 5 years). The patient has typical WBS behavior and facies, as seen in Figure 2, besides constipation, scoliosis, enuresis, precocious puberty, and mental retardation. Pituitary microadenoma was diagnosed at 11 years of age. The second patient (Figure 2(b)) was a boy, born at term, cyanotic, with no available information regarding weight and height at birth. He had neuromotor development delay (sat at 2 years, walked at 3 years, and spoke at 2 years of age) and showed typical WBS facies and behaviour, hypothyroidism, unilateral radioulnar synostosis, and bladder diverticulum.

The most prevalent clinical characteristics of the 55 patients are shown in Table 1. These included typical WBS facies (98.2%), developmental delay (98.2%), hypersocial behavior (94.5%), hyperacusis (94.5%), and congenital heart disease (81.2%).

Congenital heart disease was present in 45/55 patients, and SVAS was the most prevalent type (19/45 or 42.2% of the cases). Isolated SVAS was present in 12 patients; SVAS was associated with other cardiac anomalies in 7 patients. Three patients had echocardiogram reports of aortic stenosis, but it was not supravalvular. Pulmonary stenosis was the second most frequent abnormality, detected in 12/45 (26.7%) of the patients. Other cardiac anomalies were found in the other patients (14/45), either in isolation or in combination, as follows: mitral valve prolapse, aortic coarctation, pulmonary artery stenosis, interatrial septal defect, ventricular septal defect, tricuspid insufficiency, pulmonary valve insufficiency, mitral valve insufficiency, and bicuspid aortic valve.

One patient with SVAS that was surgically repaired in childhood presented with congestive heart failure at 19 years of age and needed a heart transplant. However, she died due to CMV infection on the 30th day after transplant.

Two other patients, a 13-year-old girl and a 19-year-old boy, died due to cardiovascular complications.

Ten out of 55 patients (18.2%) did not have congenital heart disease.

Short stature was present in 24 patients (12 females and 12 males). Microcephaly was present in 17 patients, 13 of whom were females.

Hypercalcemia was detected in only one patient, at 1.6 years of age. Another patient had serum calcium in the upper limit of normal. Two other patients presented with nephrocalcinosis, and one presented with hypercalciuria but normal serum calcium.

Noncongenital hypothyroidism was diagnosed in 8 patients (14.5%). Seven patients (7.3%) had subclinical hypothyroidism. None of the patients had abnormal fasting blood glucose levels.

Strabismus was present in 19 patients (34.5%), and hernias, either umbilical or inguinal, were present in 20 patients (36.4%). Three patients (5.4%) presented with lacrimal duct obstruction.

Genitourinary symptoms, including mainly urinary urgency and nocturnal enuresis, were reported in 45 patients (85.4%).

Twenty patients (36.4%) presented with arterial pressures above the normal levels for their age, gender, and height percentiles. The ages of these patients ranged from 4 to 23 years old. Four of them (20% of the patients with hypertension) had renal artery stenosis. Three (aged 7–13 years old) underwent corrective surgery, and one is awaiting the intervention. One patient required 2 procedures at 8 and 11 years of age.

Scoliosis was present in 31 of the 55 patients (56.4%), and radioulnar synostosis was present in 6 (10.9%).

All patients had delays in at least one motor milestone and/or had mental retardation. One patient was diagnosed with panic disorder, and one had visual hallucinations.

Some clinical manifestations rarely described in WBS were found in our patients as follows: vertebral fusion (2 patients with cervical vertebral fusion and another with lumbosacral vertebral fusion), accessory spleen (*n* = 1), scrotal nodule (*n* = 1), labia majora hypertrophy (*n* = 1), sagittal craniosynostosis (*n* = 1), neonatal tooth (*n* = 1), and muscular hernia in the leg (*n* = 1).

## 4. Discussion

For geneticists, WBS is a well-known syndrome. It is usually promptly recognizable by the characteristic facial dysmorphisms and typical hypersocial behavior. Severe cases of neonatal hypercalcemia can result in death before a diagnosis of WBS has been considered. For this reason, neonatologists should consider WBS diagnosis in neonates with hypercalcemia and/or intrauterine restriction and SVAS.

Regarding the frequency of congenital heart disease in this cohort (81.8%) and the most prevalent type, SVAS (42.2%), the data are concordant with the literature [2]. A significant number of the patients were referred to our service from the WBS National Patient Association (ABSW), not from the cardiology department of the hospital. This could have contributed to the SVAS frequency being below 50%. Although it is an important sign of the syndrome, SVAS is not pathognomonic. It is important to reinforce that the absence of congenital heart disease does not rule out WBS diagnosis. Because cardiovascular disease in WBS has been credited to the* ELN* gene deletion, it is intriguing that at least 15% of the patients with this deletion do not have cardiac abnormalities. Epigenetic factors such as copy number variation in other regions of the genome might play a role.

Sudden death is one of the complications in WBS [31–35]. Some necropsy cases revealed stenoses of the coronary arteries and severe biventricular obstruction with myocardial ischemia, decreased cardiac output, and arrhythmias as causes of death [31]. Other patients died after anesthetic procedures [36]. A phenomenon called Kounis syndrome can occur when inflammatory mediators, possibly due to massive mast cells degranulation, induce coronary spasm or obstruction in patients with preexisting coronary disease [37, 38]. We had 3 patients that died during adolescence due to cardiovascular complications. One underwent heart transplantation; the indications and outcome of this procedure in WBS are scarce in the literature.

The prevalence of other findings in WBS in this cohort is the same as reported by other groups in regard to facial dysmorphisms, hypersocial behavior, neuromotor delay, hyperacusis, short stature, and microcephaly [39]. However, only one patient presented with hypercalcemia, a feature that is usually linked to WBS because of its description. Hypercalcemia often manifests in the first years of life [2], and some of our patients might have presented this abnormality before the diagnosis of WBS was considered. Because serum calcium is not routinely measured in the neonate unit or the pediatric ER and most of our patients have not been diagnosed with WBS by this time, this may be a bias in our cohort. In addition, hypercalcemia can present at any time in a WBS patient's life; thus, although our patients did not have hypercalcemia during the assessment period, they are still at risk and should be periodically monitored for calcium disturbances [39, 40].

The prevalence of thyroid abnormalities in our cohort (14.3% of hypothyroidism and 7.3% of subclinical hypothyroidism) is similar to that of other studies, even though there is a wide range of the reported prevalences (2–38%) [39, 41]. Because hypothyroidism can aggravate some of the clinical manifestations of WBS and is a treatable condition, periodic monitoring of thyroid hormone levels in WBS patients is recommended. Currently, there is a recommendation of assessment every two years (American Academy of Pediatrics, 2001); however, in our protocol, we tested yearly, and several cases were diagnosed; thus, the interval of testing should be shorter.

Diabetes mellitus is a well-described late manifestation of WBS [40]. Although none of our patients had abnormal fasting glucose levels, most of them were children and adolescents; diabetes in WBS is more common in the adult population [40, 42].

The frequencies of other manifestations in this cohort, such as strabismus, hernias, scoliosis, and radioulnar synostosis, were concordant to the prevalence and variations described in literature [16, 39, 43, 44].

On the other hand, urinary problems were somewhat more frequent in this cohort (85.4% in our study versus 68% in the literature) [16, 39]. This could be due to the fact that a urologist evaluated all of our patients.

Arterial hypertension was detected in 20/55 patients (36.4%), one of whom was diagnosed at 4 years old; this can be an early complication in WBS. In this syndrome, there is a lifetime risk of developing arterial hypertension of 50% [45], and this has been reported even in 1-month-old patients [46]. Four out of 20 patients in our group (20%) had renovascular disease. Arterial hypertension due to renal artery stenosis is described in 44% of WBS patients [47]. Thus, every patient with WBS, regardless of age, should be monitored for blood pressure (American Academy of Pediatrics, 2001), and, in the case of hypertension (using appropriate curves for age and height percentiles), evaluation of the renal arteries is mandatory.

The occurrence of other rare findings in our patients (e.g., accessory spleens, neonatal tooth, and muscle hernias) and their relation to WBS could not be determined because the prevalence of each finding separately was low. Lacrimal duct stenosis and craniosynostosis, although uncommon, were already described in WBS [48, 49]. One patient presented with recurrent patellar dislocation, which has also already been described in WBS [50].

FISH has been the gold-standard method for the diagnosis of WBS. Among the 55 patients studied by MLPA in this study, 16 also had positive FISH results. However, two patients with typical physical and behavioral characteristics of WBS had negative FISH results but positive polymorphic marker analysis and MLPA results, which detected the typical deletion. After the positive results in both cases using MLPA method (deletion of all the probes, not an atypical deletion), we contacted the laboratory that had performed FISH. The FISH tests were repeated and the results were positive for the microdeletion. This emphasizes the importance of testing with another method or repeating the test when clinical and laboratory analyses diverge. A group from Netherlands studied 63 patients by FISH and MLPA. In 53/63 patients, the microdeletion was detected by both methods. In 10 patients, the results were negative with MLPA and FISH. However, one patient with a small, atypical microdeletion could only be diagnosed with MLPA; FISH using commercial probes was negative [51]. Thus, the gold-standard test to the diagnosis of WBS should be revised.

MLPA is also used to diagnose many other syndromes of microdeletion and microduplication, such as Smith-Magenis, DiGeorge, Alagille, Prader-Willi, and Angelman syndrome. There are specific kits for each syndrome or kits with a few probes of multiple syndromes (e.g., kits to diagnose some mental retardation syndromes). MLPA has also been proven useful for prenatal diagnosis using amniotic fluid for microdeletion and microduplication syndromes and for the diagnosis of trisomies [52–55].

## 5. Conclusions

The assessment and long follow-up of WBS patients by several medical specialties is of great relevance due to the relatively high prevalence of multisystem manifestations and complications.

MLPA was effective in confirming the diagnosis of WBS and can be used as the first exam in developing countries due to its lower cost compared with FISH. In addition, MLPA has the advantage of detecting atypical deletions and can be useful when FISH is negative in patients with clinical characteristics that are highly suggestive of WBS.

## Figures and Tables

**Table 1:** Clinical characteristics of WBS patients.

Clinical characteristic	*n*	%
Typical WBS facies^*^	54/55	98.2
Developmental delay	54/55	98.2
Hypersocial behavior	52/55	94.5
Hyperacusis	52/55	94.5
Congenital heart disease	45/55	81.2
Genitourinary symptoms	47/55	85.5
Short stature	24/55	43.6
Hypertension	20/55	36.4
Microcephaly	17/55	31.0

^∗^Typical WBS facies based on the score proposed by the Genetics Committee of the American Academy of Pediatrics (2001).
